# Near-infrared fluorescence imaging and photodynamic therapy with indocyanine green lactosomes has antineoplastic effects for gallbladder cancer

**DOI:** 10.18632/oncotarget.27193

**Published:** 2019-09-24

**Authors:** Hidehiko Hishikawa, Masaki Kaibori, Takumi Tsuda, Kosuke Matsui, Tadayoshi Okumura, Eiichi Ozeki, Kengo Yoshii

**Affiliations:** ^1^ Department of Surgery, Kansai Medical University, Hirakata, Osaka, Japan; ^2^ Technology Research Laboratory, Shimadzu Corporation, Kyoto, Japan; ^3^ Department of Mathematics and Statistics in Medical Sciences, Kyoto Prefectural University of Medicine, Kyoto, Japan

**Keywords:** near infrared fluorescence imaging, photodynamic therapy, indocyanine green, lactosome, gall bladder cancer

## Abstract

**BACKGROUND:** The diagnostic use and therapeutic effect of near infrared fluorescence (NIF) imaging and photodynamic therapy (PDT) was investigated for gallbladder cancer using indocyanine green (ICG)-lactosomes.

**RESULTS:** PDT was toxic for NOZ cells treated with ICG-lactosomes. Fluorescence intensity in the tumor region of mice administered ICG-lactosomes, but not ICG alone, was higher than the healthy contralateral region ≥24 hours after injection. PDT exerted immediate and continuous phototoxic effects in NOZ implanted mice injected with ICG-lactosomes. Enhanced antitumor effects were observed in the twice irradiated group compared with the once irradiated group.

**METHOD:** ICG or ICG-lactosomes were added to the human gallbladder cancer cell line NOZ followed by PDT and cell viability was measured. Mass spectrometry of ICG and ICG-lactosomes was performed after PDT. ICG or ICG-lactosomes were intravenously administered to BALB/c nude mice implanted subcutaneously with NOZ cells and fluorescence was evaluated by NIF imaging. Implanted tumors underwent PDT and antitumor effects were analyzed after performing irradiation once or twice in ICG-lactosome groups.

**CONCLUSIONS:** ICG-lactosomes accumulated in xenograft tumors and PDT had an antitumor effect on these malignant tumors. NIF imaging with ICG-lactosomes and PDT may be useful diagnostic and/or therapeutic agents for gallbladder cancer.

## INTRODUCTION

Gallbladder cancer is difficult to detect at an early stage, and it is often advanced at the time of discovery. The 5-year survival rate is less than 30% in all stages, less than 20% in stage III, around 3% in stage IV, and it has a much poorer prognosis than other carcinomas. This is because metastasis to the lymph nodes and liver, as well as peritoneal seeding occurs early. Treatment of gallbladder cancer mainly consists of surgical therapy, chemotherapy, and radiotherapy. Surgery is the first-choice treatment. The response rate of chemotherapy in unresectable cases is 21%–38% [1], and radiation therapy is a palliative treatment.

The photosensitizer indocyanine green (ICG) is a water-soluble tricarbocyanine dye that emits fluorescence at a peak wavelength of 830 nm when irradiated with near-infrared excitation light [2]. In clinical practice, ICG is used to identify sentinel lymph node metastasis in breast cancer, as well as ophthalmologic angiography and coronary blood flow assessment. In the field of gastrointestinal surgery, it is used for the local diagnosis of intraoperative hepatocellular carcinoma (HCC) by liver function evaluation and fluorescence imaging [2–5]. Recently, intravenous ICG administration was performed during surgery to evaluate blood flow. ICG has also been applied to photodynamic therapy (PDT) using a photochemical reaction between the photosensitizer and laser light of a specific wavelength. Briefly, following the administration of a photosensitizer, it accumulates in cancer tissues, and then the tumor is irradiated with near infrared light. The activated photosensitizer reacts with endogenous oxygen, resulting in the production of singlet oxygen that induces cell death such as apoptosis in tumor cells. The mechanisms of tumor suppression by PDT using ICG-lactosomes involves singlet oxygen and ICG degradation products generated by photochemical reactions. When a photosensitizer is exposed to light of a particular wavelength during PDT, it is activated from the ground state to the excited state. When it returns to the ground state, it releases energy that is transferred to molecular oxygen to generate reactive oxygen species (ROS) such as singlet oxygen and free radicals. These ROS mediate cytotoxicity. Similarly, singlet oxygen can be generated when ICG is exposed to near infrared excitation light. ICG is degraded by singlet oxygen itself and degradation products further reduce cell viability [4, 6]. Furthermore, heat is generated by the reaction, and this thermal effect also contributes to the tumor suppressing effect [4]. PDT is mainly applied as a topical therapy for malignant tumors such as skin cancer [7] and superficial bladder cancer [8]. In addition, PDT has recently been widely accepted as a treatment option for early gastric, esophageal, and lung cancers [9].

Polymer micelle nanocarriers might have potential in tumor imaging and anti-tumor therapy. These micelles accumulate in solid tumors because of their enhanced permeability and retention (EPR) effects, whereby 30–100 nm nanoparticles passively accumulate because of the high permeability of tumor blood vessels and reduced lymph flow around the tumor tissue [10]. However, nanocarriers are trapped by cells of the hepatic reticuloendothelial system (RES) and may accumulate in the liver. Lactosomes are a core-shell type nanocarrier, a polymeric micelle composed of an amphiphilic polydepsipeptide with a hydrophobic block of helical poly (L-lactic acid) (PLLA) and a hydrophilic block of poly (sarcosine). Lactosomes, a biocompatible, biodegradable substance that does not induce acute toxicity, selectively accumulated in liver tumors and avoided the RES [11–14]. In recently published research, the authors of this study demonstrated that ICG-lactosomes accumulated in xenograft HCC tumors, and that PDT had antineoplastic effects on these malignant implants. Near infrared fluorescence (NIF) imaging and PDT with ICG-lactosomes might be useful diagnostic and/or therapeutic strategies for HCC [15].

In this study, a polymeric micelle nanocarrier termed “ICG-lactosomes” that accumulated in a tumor-specific manner was prepared, and its diagnostic usefulness for gallbladder cancer and the therapeutic effect of PDT were examined.

## RESULTS

### *In vitro* PDT

**Figure 1:**
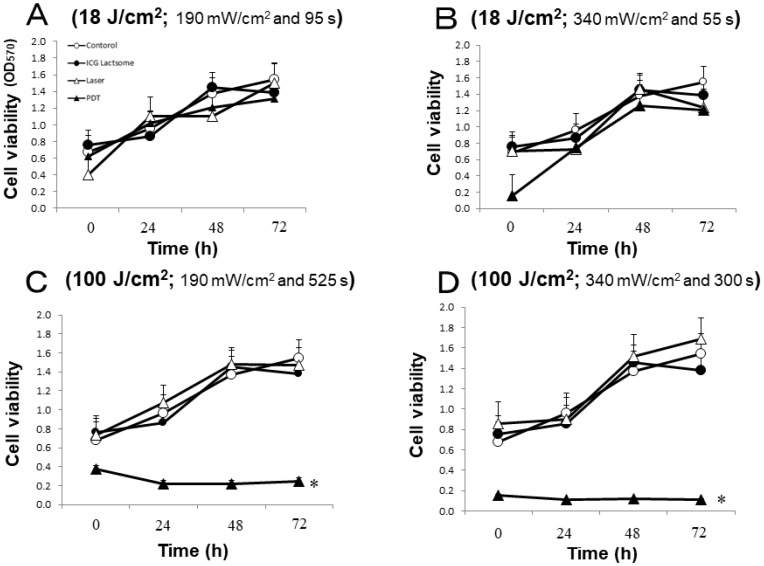
Effect of PDT on cell viability in ICG-lactosomes-treated NOZ cells. Cells were divided into four groups: control, ICG-lactosomes, laser, and PDT (ICG-lactosomes + laser). The laser and PDT groups were irradiated with (**A**) 18 J/cm^2^ (190 mW/cm^2^ and 95 s), (**B**) 18 J/cm^2^ (340 mW/cm^2^ and 55 s), (**C**) 100 J/cm^2^ (190 mW/cm^2^ and 525 s), or (**D**) 100 J/cm^2^ (340 mW/cm^2^ and 300 s). Cell viability after irradiation was measured by MTT assay at the indicated times. The results are presented as the mean ± SEM (*n =* 4 dishes/time/group). ^*^ *P* < 0.001, PDT vs other groups.

The effect of laser irradiation with low or high fluence (18 or 100 J/cm^2^, respectively) on cell viability in ICG-lactosome-loaded NOZ cells was examined. The cells were divided into four groups: control, ICG-lactosomes, laser (laser without ICG-lactosomes) and PDT (ICG-lactosomes + laser). The effects of laser irradiation with different fluence rates (18 J/cm^2^ or 100 J/cm^2^) and times were examined. The irradiation conditions were as follows: (A) 18 J/cm^2^ (190 mW/cm^2^ and 95 s); (B) 18 J/cm^2^ (340 mW/cm^2^ and 55 s); (C) 100 J/cm^2^ (190 mW/cm^2^ and 525 s); and (D) 100 J/cm^2^ (340 mW/cm^2^ and 300 s). At 72 hours post irradiation, cell viability was not reduced in any group under the 18 J/cm^2^ condition (Figure 1A and 1B). However, it was significantly reduced in the PDT group (closed triangles) under the 100 J/cm^2^ condition (Figure 1C and 1D).

**Figure 2:**
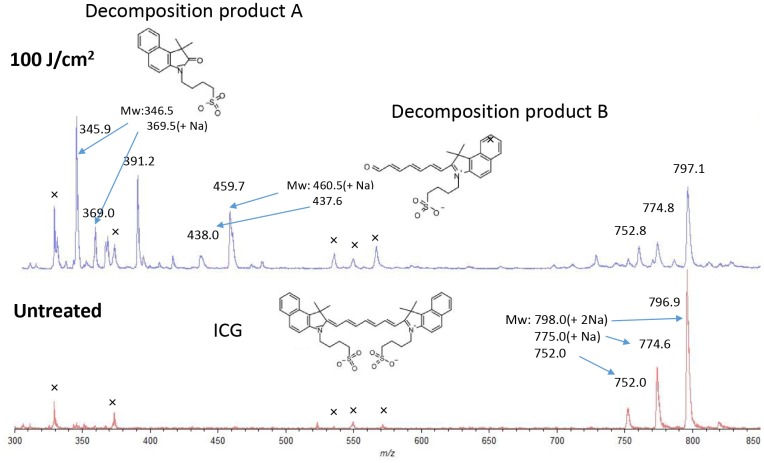
Matrix assisted laser desorption/ionization time of flight mass spectrometry of ICG in water with or without laser irradiation. During laser treatment (100 J/cm^2^, 340 mW/cm^2^ and 300 s), ICG is cleaved to decomposition products A and B by oxidation (upper).

Decomposition was confirmed using Matrix Assisted Laser Desorption/Ionization time of flight mass spectrometry (MALDI-TOF-MS) by irradiating the ICG aqueous solution with a laser (Figure 2). After irradiation (100 J/cm^2^, 340 mW/cm^2^ and 300 s), two decomposition products, A (*m/z* 346.0, 368.8(+Na)) and B (*m/z* 438.0, 459.7(+Na)), derived from cleavage of the double bond by oxidation in ICG (752.0, 774.6(+Na), 798.0(+2Na)) were observed [4]. These decomposition products were not near infrared fluorescent substances.

### *In vivo* imaging

**Figure 3:**
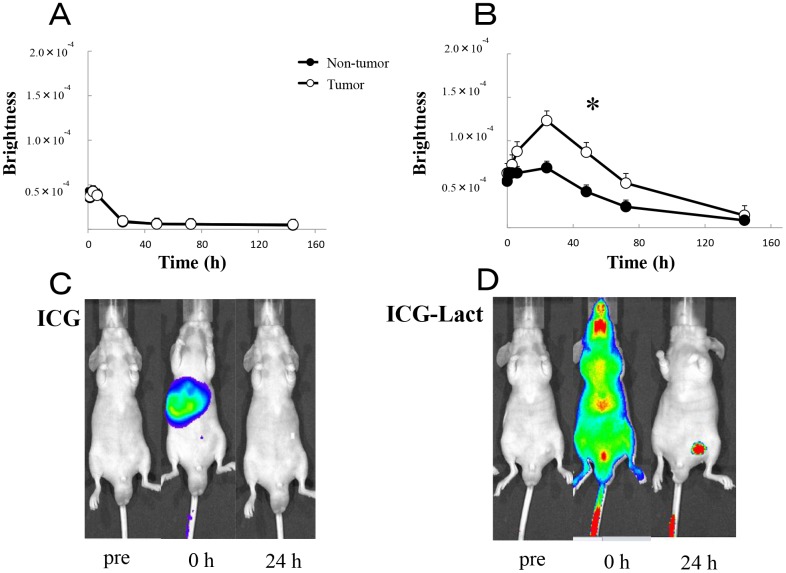
*In vivo* fluorescence imaging in ICG and ICG-lactosomes mice with subcutaneous tumors. Fluorescence imaging was acquired using an IVIS system in ICG-treated mice (ICG, **A** and **C**) and ICG-lactosomes-treated mice (ICG-Lact, **B** and **D**). After injecting ICG or ICG-lactosomes, brightness of the tumor (open circles) and non-tumor (contralateral inguinal, closed circles) areas were measured at the indicated times. ^*^ *P* < 0.05 between tumor and non-tumor regions in the ICG-lactosomes group. The results are presented as the mean ± SEM (*n =* 5/group).

Using an IVIS system, *in vivo* fluorescence imaging was compared between the ICG-treated and ICG-lactosome-treated mice with subcutaneous tumors. In the ICG-treated animals, ICG fluorescence accumulated in the liver immediately after the injection and migrated from the liver to the intestinal tract via bile duct exclusion within 1–6 hours (Figure 3A and 3C). After 24 hours, most of the ICG fluorescence was excluded from the body, although a minor portion remained in the tumor and normal tissue areas. Comparison of the brightness of tumor regions with that of normal tissue (contralateral inguinal) regions indicated no significant difference (Figure 3A).

However, in the ICG-lactosome-treated animals, the fluorescence was distributed throughout the whole body immediately after the injection (Figure 3D). After 24–72 hours, high levels of ICG fluorescence had accumulated and remained in the tumor area, and there was a significant difference between the tumor and contralateral inguinal regions (Figure 3B and 3D). After 1 week, ICG fluorescence was still observed in the tumor area, while no fluorescence existed in the surrounding tissues or other organs (data not shown).

### *In vivo* PDT

**Figure 4:**
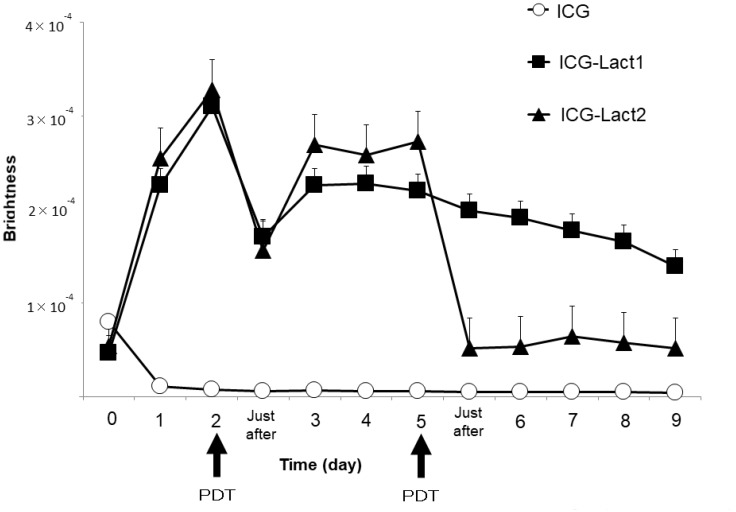
Re-accumulation of fluorescence imaging to the tumor site after PDT in mice administered ICG-lactosomes. Accumulation of fluorescence imaging in mouse subcutaneous tumors after ICG or ICG-lactosomes administration was observed daily using IVIS. On the second day of administration, PDT irradiation (1150 mW/cm^2^, 90 s (100 J/cm^2^)) was performed in the ICG (ICG (*n =* 5), open circles) and ICG-lactosomes-once-irradiated (ICG-Lact1 (*n =* 5), closed squares) groups. The second irradiation was performed on day 5 after the first PDT in the ICG-lactosomes-twice-irradiated group (ICG-Lact2 (*n =* 5), closed triangles). The results are presented as the mean ± SEM.

Using the IVIS system, the fluorescence intensity of the tumor site after PDT was compared among the ICG, ICG-lactosomes-once-irradiated and twice-irradiated groups (Figure 4). In mice to which ICG-lactosomes were administered, a decrease in the fluorescence intensity of the tumor site was confirmed immediately after laser irradiation (PDT, day 2), but a re-increase in the fluorescence intensity was observed 3 days after the first irradiation (ICG-lactosomes-once-irradiated group (ICG-Lact1), closed squares). Then, the fluorescence intensity of the tumor site decreased gradually. In the ICG group, no increase in the fluorescence intensity at the tumor site was observed (ICG, open circles). In the ICG-lactosomes-twice-irradiated group (second PDT, day 5), no repopulation of the tumor site was observed thereafter (ICG-Lact2, closed triangles).

**Figure 5:**
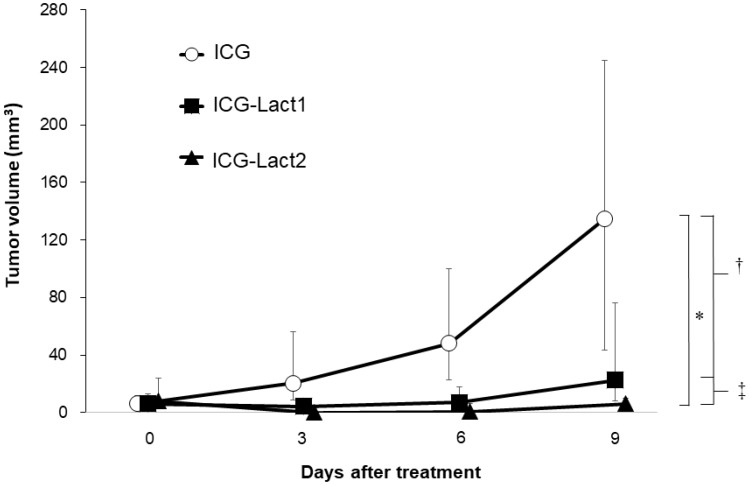
Effect of PDT on tumor growth in ICG and ICG-lactosomes mice with subcutaneous tumors. On the second day of administration, PDT (1150 mW/cm^2^, 90 s (100 J/cm^2^)) was performed in the ICG (ICG (*n =* 8), open circles) and ICG-lactosomes-once-irradiated (ICG-Lact1 (*n =* 8), closed squares) groups. The second PDT was performed on day 5 after the first PDT in the ICG-lactosomes-twice-irradiated group (ICG-Lact2 (*n =* 8), closed triangles). The tumor volumes were measured at the indicated times. ^*^ *P* < 0.001 between the ICG and ICG-Lact2 groups. ^†^ *P* < 0.05 between the ICG and ICG-Lact1 groups. ^‡^ *P* < 0.05 between the ICG-Lact1 and ICG-Lact2 groups. The results are presented as the mean ± SEM (*n =* 8/group).

The anti-tumor effect was compared among the ICG, ICG-lactosomes-once-irradiated and ICG-lactosomes-twice-irradiated groups (Figure 5). The median tumor volume of the ICG group increased from day 0 (6.0 mm^3^ [4.0–18.0 mm^3^] to 9 (135.0 mm^3^ [22.5–294.0 mm^3^], open circles). In contrast, the median tumor volume of the ICG-lactosomes-once-irradiated (ICG-Lact1, day 0 (6.0 mm^3^ [0.5–18.0 mm^3^] and day 9 (22.5 mm^3^ [6.0–125.0 mm^3^], closed squares) and twice-irradiated (ICG-Lact2, day 0 (8.0 mm^3^ [1.0–40.0 mm^3^] and day 9 [6.0 mm^3^ (0.0–13.5 mm^3^), closed triangles) groups showed no significant growth from days 0 to 9 compared with the ICG group. However, there was a significant difference in the mean tumor volume between the ICG and ICG-Lact1/ICG-Lact2 groups (*P* < 0.001 and *P* < 0.05, respectively). Furthermore, there was a significant difference in the mean tumor volume between the two ICG-lactosomes groups (*P* < 0.05).

**Figure 6:**
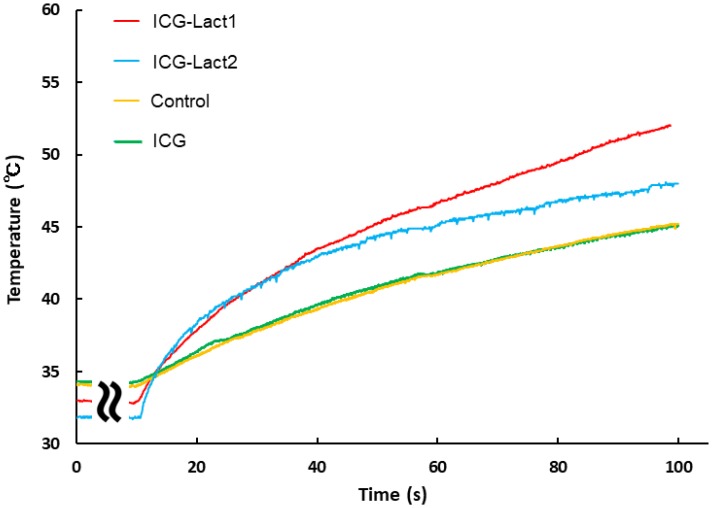
Effect of PDT on tumor temperature in ICG and ICG-lactosomes mice with subcutaneous tumors. On the second day of administration, PDT (1150 mW/cm^2^, 90 s (100 J/cm^2^)) was performed in the control (without ICG and ICG-lactosomes, yellow), ICG (green) and ICG-lactosomes-once-irradiated groups (ICG-Lact1, red). The second PDT was performed on day 5 after the first PDT in the ICG-lactosomes-twice-irradiated group (ICG-Lact2, blue). After the first or second PDT, the temperature of the tumor area was measured at the indicated times. Representative results of three independent experiments are shown.

During the laser irradiation, changes in temperature of the tumor area were similar between the control (yellow) and ICG administration (green) groups (Figure 6). The mean (standard deviation) temperature after 100 s was as follows: Control group (*n =* 5), 43.9° C (1.18); ICG group (*n =* 4), 43.5° C (0.99); ICG-Lact1 group (*n =* 4), 52.0° C (1.18); and ICG-Lact2 group (*n =* 2), 48.9° C (1.27). The change in temperature in the ICG-lactosomes-once-irradiated group (ICG-Lact1, red) was the highest, followed by the twice-irradiated group (ICG-Lact2, blue). The changes in temperature in the two ICG-lactosomes groups were higher than those in the control and ICG groups.

**Figure 7:**
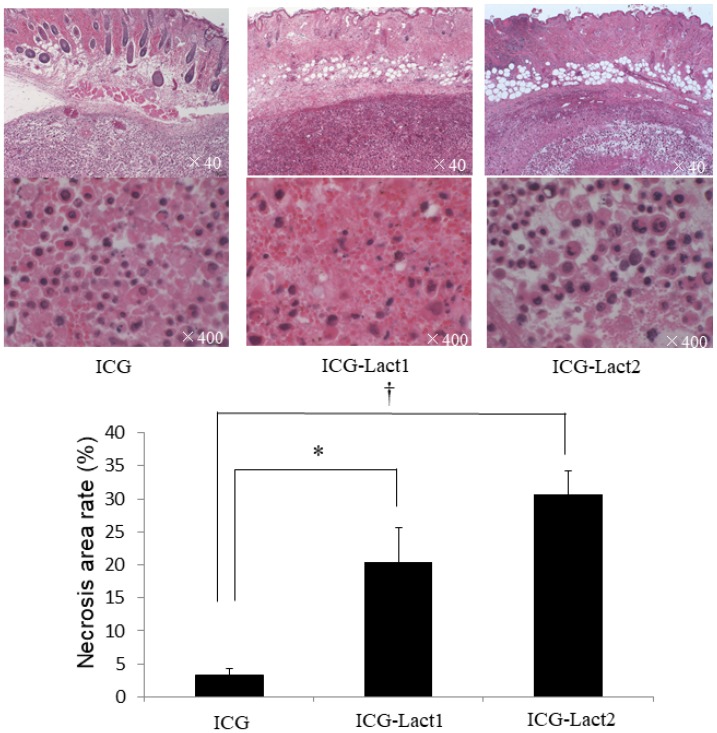
Effect of PDT on necrosis of subcutaneous tumors in the ICG and ICG-lactosomes mice. Tumors were obtained for the examination of necrosis 24 hours after irradiation (1150 mW/cm^2^ and 90 s, (100 J/cm^2^)) in ICG, ICG-lactosomes-once-irradiated and ICG-lactosomes-twice-irradiated groups. In the three groups, the percentage of necrotic area in the tumor was calculated as the necrosis rate. ^*^ *P* < 0.05 between the ICG and ICG-lactosomes-once-irradiated groups. ^†^ *P* < 0.001 between the ICG and ICG-lactosomes-twice-irradiated groups. The results are presented as the mean ± SEM (*n =* 5/group).

Histopathology revealed that the necrosis rates in the ICG-lactosomes-once and twice-irradiated groups were significantly higher than those in the ICG group (Figure 7). The distribution of necrosis was homogenous in tumors among the three groups, and it was dominant in areas near to the irradiation site.

## DISCUSSION

This research demonstrated that ICG-lactosomes accumulated in xenograft tumors and that PDT had an antitumor effect on malignant tumors. Funayama et al. reported that ICG-lactosomes selectively accumulated in spinal cord metastasis and PDT using ICG-lactosomes delayed the onset of paralysis in a rat spinal cord metastasis model [16–18]. Hirano et al. reported a comparison of the diagnostic and therapeutic effects of ICG and ICG-lactosomes [6]. ICG-lactosomes selectively accumulated in lymph node metastasis and peritoneal dissemination of gastric cancer, although their accumulation in tumors was not observed in the ICG group. PDT using ICG-lactosomes significantly suppressed the growth of metastasis compared with PDT using ICG.

In this study, *in vivo* imaging demonstrated that ICG-lactosomes, but not ICG alone, were specifically incorporated into, and clearly visualized in, subcutaneous xenograft tumors of human gallbladder cancer. Furthermore, it was found that PDT using ICG-lactosomes induced necrosis and suppressed tumor growth *in vitro* and *in vivo*. In the present study, a decrease in the fluorescence intensity of the tumor site immediately after laser irradiation was confirmed, and a re-increase in the fluorescence intensity was observed 3 days after the first irradiation in mice to which ICG-lactosomes were administered. In the MALDI-TOF-MS study, it was found that the double bond in the ICG molecule was cleaved by oxidation using laser irradiation in water [4]. This indicates that a resonant structure in ICG was broken down and therefore, near infrared fluorescence was lost in the tumor site after laser irradiation. Furthermore, ICG-lactosomes in the blood, which are not decomposed by laser irradiation, were administered to tumor sites by the EPR effect after the first irradiation.

A second PDT can be performed by repopulating the tumor. Double irradiation had a greater antitumor effect compared with a single irradiation. In the future, it may be possible to apply continuous treatment by determining the optimal dose and number of doses of PDT. The anti-tumor effects of PDT were not obtained in the ICG group because of the insufficient accumulation of ICG in the tumor. During deep cancer treatment, PDT combined with ICG, a near-infrared photosensitizer, has advantages compared with PDT combined with an ultraviolet photosensitizer. However, ICG concentrations in the plasma following intravenous administration were decreased rapidly (half-life in the blood is 3–4 min). Therefore, ICG cannot be efficiently accumulated in tumors. However, the attenuation constant (half-life in the blood) of ICG-lactosomes was calculated to be 0.042 h^−1^ (24 hours) [13]. Lactosomes had accumulated at the tumor region 48 hours after their administration.

The heat generated by photothermal reactions is also involved in the mechanisms of tumor suppression by PDT using ICG-lactosomes. The authors of this study previously demonstrated that temperature measurements during *in vivo* PDT using ICG-lactosomes were increased by 50° C or more in hepatocellular carcinoma [15]. The current study also showed that heat required to damage tumor cells was higher than 50° C at the time of single irradiation and 45° C at the time of double irradiation, indicating it was involved in tumor growth suppression. In addition to temperature control, it is necessary to optimize the ICG-lactosomes dose as well as the fluence of the laser irradiation to prevent tumor growth in the future.

This study had several limitations. First, the number of mice used in the experiments might be too low to determine statistical significance. Furthermore, it is unclear whether ICG-lactosomes might induce adverse effects when used clinically, and whether ICG-lactosomes will accumulate in the early stages of gallbladder cancer as observed for mouse subcutaneous tumors. In the future, studies on the pharmacokinetics and PDT effects of ICG-lactosomes should be conducted using large animal models.

In conclusion, this study suggests that ICG-lactosomes are useful as novel diagnostic and therapeutic agents for gallbladder cancer. In the future, this research group will optimize the dose and irradiation conditions of the drug and consider the treatment effects on other carcinomas to develop this treatment for clinical applications. PDT with ICG-lactosomes used in the present study can be repeated and be used for endoscopic and laparoscopic treatments for gallbladder cancer.

## MATERIALS AND METHODS

### Cancer cell lines

NOZ cells (a moderately differentiated human gallbladder cancer cell line) were purchased from the Japan Research BioSource (JCRB) Cell Bank (Osaka, Japan). The cells were suspended in Williams’ medium E (Wako, Osaka, Japan) and incubated at 37° C in a humidified atmosphere of 5% CO_2_ in an incubator. Penicillin (100 U/mL), streptomycin (100 mg/mL) and amphotericin B (0.25 mg/mL) (antibiotic-antifungal mixed stock solution; Nacalai Tesque, Kyoto, Japan) supplemented with Williams’ medium E was used.

### *In vitro* PDT

NOZ cells were seeded in 96-multiwell plates (BD Falcon, Tokyo, Japan) at 2 × 10^4^ cells/100 μL medium/well and incubated for 24 hours. The cells were divided into four groups: control (without ICG-lactosomes and laser irradiation), ICG-lactosomes (ICG-lactosomes without laser irradiation), laser (laser without ICG-lactosomes) and PDT (ICG-lactosomes + laser). Phosphate buffered saline (PBS (-)) containing 2 mg/mL ICG-lactosomes (containing 0.2 mg/mL ICG) was used in the ICG-lactosomes and PDT groups, and PBS (-) was used for control and laser groups. The irradiation conditions were classified into the following four categories: (A) 18 J/cm^2^ (190 mW/cm^2^ and 95 s); (B) 18 J/cm^2^ (340 mW/cm^2^ and 55 s); (C) 100 J/cm^2^ (190 mW/cm^2^ and 525 s); and (D) 100 J/cm^2^ (340 mW/cm^2^ and 300 s). Lasers and PDT groups were irradiated immediately after changing the medium using a near infrared (810 ± 10 nm) laser source (AVL-20, Japan, Kyoto, Japan). The fluence rate and irradiation time of the laser were set by either method as 190 mW/cm^2^ and 95 s, 340 mW/cm^2^ and 55 s, 190 mW/cm^2^ and 525 s, or 340 mW/cm^2^ and 300 s corresponding to a fluence of about 18 J/cm^2^ or 100 J/cm^2^. The laser probe was set 2 cm above the plate. After irradiation, each well was replaced with fresh medium and incubated for 0, 24, 48 and 72 hours.

### MALDI-TOF-MS

ICG and decomposition products were analyzed by AXIMA Performance (Shimadzu Corporation, Japan) using α-cyano-4-hydroxy cinnamic acid in H_2_O: CH_3_CN (1:1) as a matrix.

### Evaluation of cell viability after irradiation

Cell morphology was observed with a phase contrast microscope and the survival rate after irradiation was measured by MTT assay (Cayman Chemical, Ann Arbor, MI, USA). Then, 10 μL of MTT reagent was added to each well and incubated at 37° C for 3 hours in a CO_2_ incubator. Then, the medium was aspirated, 100 μL of crystal dissolved solution was added to dissolve the formazan crystals, and cell viability was determined using a microplate reader at a wavelength of 570 nm.

### Animal models

Male BALB/c nude mice (5-weeks old) were purchased from Shimizu Laboratory Co., Ltd. (Kyoto, Japan). Mice were kept at 22° C under a 12-hour/12-hour light/dark cycle and given food and water *ad libitum*. All animal experiments were conducted according to guidelines for the care and use of laboratory animals of the National Institutes of Health and approved by the Animal Care Committee of Kansai Medical University (No. 18-072). Mice were anesthetized with isoflurane and injected subcutaneously into the left inguinal region with 5 × 10^6^ NOZ cells in 100 μL culture medium. When tumors reached 10–100 mm^3^ (approximately 3 weeks after transplantation), the *in vivo* imaging or PDT experiment was terminated.

### *In vivo* imaging and PDT

Model mice were anesthetized with isoflurane and intravenously injected through the tail vein with 100 μL of 20 mg/mL ICG-lactosomes containing 0.2 mg/mL ICG (ICG-lactosomes group, *n =* 5) or 100 μL of 0.2 mg/mL ICG (ICG group, *n =* 5) dissolved in saline. Following the administration of ICG-lactosomes or ICG, mice were anesthetized with isoflurane and imaged at 0, 3, 6, 24, 48, 72 and 150 hours. *In vivo* fluorescence imaging was performed using an IVIS system (PerkinElmer, Waltham, MA, USA). The transplanted tumor was illuminated with 780 nm excitation light and fluorescence was obtained using an 845-nm filter. The luminance of the tumor and non-tumor (contralateral inguinal) regions was measured and recorded from the obtained fluorescence image.

For PDT, the model mice were anesthetized and ICG-lactosomes and ICG groups (*n =* 8/group) were prepared as described above. Irradiation with a near infrared laser source (AVL-15 Asuka Medical, Kyoto, Japan) was performed 48 hours post injection and a fiber probe output was placed 1 cm above the xenograft tumor. The fluence rate was 1150 mW/cm^2^ and the irradiation time was 90 s (100 J/cm^2^). Compared with the *in vitro* settings, the fluence rate was increased to 1150 mW/cm^2^. It was not possible to obtain a sufficiently high temperature increase with low fluence, and a setting was made at the maximum amount that did not cause burns to normal skin by carrying out preliminary experiments. ICG-lactosomes re-accumulated on the third day after PDT. The ICG-lactosomes group was irradiated once (ICG-Lact1, *n =* 8) or twice (ICG-Lact2, *n =* 8). In the twice irradiated group, the second PDT was performed using the same conditions as for the first PDT (1150 mW/cm^2^ and 90 s (100 J/cm^2^)) on the third day after the first irradiation (double PTD; days 2 and 5). After PDT, the tumor volume was measured every 3 days until day 9. The tumor volume was calculated by the equation (major axis × minor axis^2^)/2. The tumor temperature of PDT-treated mice was measured with a thermo camera (testo 890, Testo K. K., Japan).

### Pathology

Tumors were obtained in the ICG group and ICG-lactosomes groups (ICG-Lact1, ICG-Lact2) after irradiation. Pathological specimens were collected and necrosis examination was performed using HE staining. The necrotic area was calculated as the cumulative area within the tumor nodule. The percentage of necrotic area in the tumor was calculated as the necrosis rate.

### Statistical analysis

Data are presented as the median with range. Differences between groups were assessed by two-way repeated measures of the analysis of variance and Tukey–Kramer post hoc test. The necrosis index was assayed by the Tukey–Kramer test. The tumor volume after log converting as a variance was analyzed if the difference between groups was large. The significance level was set at *P*<0.05. All statistical analyzes were performed in R version 3.4.3 (R for Foundation for Statistical Computing, Vienna, Austria).
